# Elite UK winter wheat cultivars differ in their ability to support the colonization of beneficial root-infecting fungi

**DOI:** 10.1093/jxb/ery136

**Published:** 2018-04-10

**Authors:** Sarah-Jane Osborne, Vanessa E McMillan, Rodger White, Kim E Hammond-Kosack

**Affiliations:** 1 Department of Biointeractions and Crop Protection, Rothamsted Research, Harpenden, Hertfordshire, UK; 2 Department of Computational and Analytical Systems, Rothamsted Research, Harpenden, Hertfordshire, UK

**Keywords:** Beneficial soil-dwelling fungi, biological control of root disease, elite UK wheat cultivars, *Gaeumannomyces hyphopodioides*, Magnaporthaceae family, *Phialophora* species, soil-borne fungi, take-all disease, *Triticum aestivum*, wheat germplasm

## Abstract

In numerous countries, *Gaeumannomyces* species, within the Magnaporthaceae family, have previously been implicated in the suppression of take-all root disease in wheat. A UK arable isolate collection (*n*=47) was gathered and shown to contain *Gaeumannomyces hyphopodioides* and an unnamed Magnaporthaceae species. A novel seedling pot bioassay revealed that both species had a similar ability to colonize cereal roots; however, rye (*Secale cereale*) was only poorly colonized by the Magnaporthaceae species. To evaluate the ability of 40 elite UK winter wheat cultivars to support soil inoculum of beneficial soil-dwelling fungi, two field experiments were carried out using a naturally infested arable site in south-east England. The elite cultivars grown in the first wheat situation differed in their ability to support *G. hyphopodioides* inoculum, measured by colonization on Hereward as the subsequent wheat in a seedling soil core bioassay. In addition, the root colonization ability of *G. hyphopodioides* was influenced by the choice of the second wheat cultivar. Nine cultivars supported the colonization of the beneficial root fungus. Our findings provide evidence of complex host genotype–*G. hyphopodioides* interactions occurring under field conditions. This new knowledge could provide an additional soil-based crop genetic management strategy to help combat take-all root disease.

## Teaser

Field-based evidence suggest that specific elite UK wheat cultivars can support natural populations of the take-all root disease-supressing fungus, Gaeumannomyces hyphopodioides, in soil under a first wheat crop

## Introduction

Take-all is a root disease, caused by the recently reclassified soil-borne ascomycete fungus *Gaeumannomyces tritici* (Walker, 1981; Hernández-Restrepo *et al.*, 2016) (previous name *Gaeumannomyces graminis* var. *tritici*), which devastates wheat production worldwide. In a first wheat crop, take-all inoculum will begin to build up in the soil and then can cause severe disease in second and subsequent wheat crops. The fungus spreads across the root surface by means of runner hyphae. Infection hyphae can subsequently invade the root and destroy the root vascular tissue (Skou, 1981), leading to the formation of black necrotic lesions that disrupt water and nutrient uptake (Pillinger *et al.*, 2005). Severe root disease causes several above-ground symptoms including stunted plants, lack of grain formation, and premature ripening of the grain, which results in a loss in both grain quality and potential yield.

Historically there has been considerable interest in the biological control of take-all disease using bacterial and fungal species naturally occurring in the soil (reviewed by Wong, 1981; Hornby *et al.*, 1998; Weller *et al.*, 2002; Cook, 2003). However, successful biological control under field conditions has often been difficult due to the heterogeneous nature of the soil environment and difficulties in establishing sufficient populations of beneficial microorganisms for consistent and effective control.

Closely related fungal species within the Magnaporthaceae family have previously been implicated in the suppression of take-all disease. For example, *Gaeumannomyces hyphopodioides* (Hernández-Restrepo *et al.*, 2016) (previous names *Phialophora radicicola*, *Phialophora* sp. lobed hyphopodia and *Gaeumannomyces graminis* var. *graminis*) occurs naturally in UK grasslands (Deacon 1973) and is known to suppress take-all disease in wheat in both glasshouse and field experiments (Speakman and Lewis, 1978; Martyniuk and Myskow, 1984; Wong *et al.*, 1996). Field trials conducted in Poland (Martyniuk and Myskow, 1984) and Australia (Wong and Southwell, 1980; Wong *et al.*, 1996) examined the effect of artificial inoculation of *G. hyphopodioides* to the soil to protect wheat crops against take-all. However, only varying success was reported. *Gaeumannomyces hyphopodioides* protects wheat roots against take-all infection by inducing host resistance (Speakman and Lewis, 1978). A related unnamed Magnaporthaceae species (Hernández-Restrepo *et al.*, 2016) has previously been isolated from fields in the UK (Ward and Bateman, 1999) and in Germany (Ulrich *et al.*, 2000), but it is not known if this species can suppress take-all disease.

In this study, we explore the effect of cereal and cultivar genotype on the root colonization ability of *G. hyphopodioides* and the related Magnaporthaceae species with the aim of understanding whether host genetics can be utilized to support natural populations of these fungal species in field soil.

The specific aims of this study were 4-fold. First, our aim was to develop a new arable-derived collection of potentially beneficial fungal root colonizers (*G. hyphopodioides* and related species) and compare this with the existing arable and grassland collection reported by Hernández-Restrepo *et al.* (2016). Secondly, we wanted to establish a seedling bioassay with artificial fungal inoculum addition under controlled environment conditions, to explore their root colonization ability on different cereal species. A range of cereal genotypes were evaluated including oats, rye, triticale, and wheat. These were included to compare levels of colonization found for both the potentially beneficial fungal species and the take-all fungus. Thirdly, we aimed to explore whether there were any differences in the ability of current commercial UK winter wheat cultivars to support populations of beneficial root colonizers in a naturally *G. hyphopodioides*-infested first wheat field site. To achieve this, a post-harvest soil core bioassay, baited with a single cultivar (Hereward), was used to gauge the amount of infective fungal inoculum. Fourthly, we wanted to investigate whether different commercial cultivars varied in their ability to be colonized by *G. hyphopodioides* in the seedling soil core bioassay. Post-harvest soil cores were baited with the same field plot cultivar and compared with the cores baited with Hereward.

In the naturally infested *G. hyphopodioides* field site, the results obtained indicate that a series of complex host–microbe interactions exist, but that certain elite wheat genotypes when grown in either a first or second rotational position lead to either medium levels or very low levels of root colonization by this beneficial species. This provides an important resource for studies into the genetic and mechanistic basis of the interaction as well as potentially providing a novel way of introducing and supporting populations of this fungus under field conditions.

## Materials and methods

### Fungal isolations

Isolates of the required species were gathered post-harvest from three commercial wheat fields and one commercial barley field across the Rothamsted Farm (Hertfordshire, UK), to create an isolate collection and for the establishment of the seedling pot bioassay. The field sites had previous histories of natural populations of *G. hyphopodioides* and related species (see Supplementary Fig. S1 at *JXB* online). Soil cores were taken (between 50 and 100 depending on field size) and baited from the four fields as described for the take-all soil core bioassay (McMillan *et al.*, 2011; Supplementary Table S1). Root pieces with subepidermal vesicles resembling previously described *G. hyphopodioides* and related species symptoms were cut as 1 cm long segments and surface sterilized for 5 min in sodium hypochlorite (1:5 dilution with sterile distilled H_2_O), triple rinsed in sterile distilled H_2_O, blotted dry on filter paper, and plated onto potato dextrose agar (PDA) (Sigma Aldrich^®^, Dorset, UK) amended with penicillin (50 µg per plate) and streptomycin (50 µg per plate). Plates were incubated at 21 °C and cultures resembling *Gaeumannomyces* species were plated onto fresh PDA amended with penicillin and streptomycin, and incubated for 2 weeks. Fungal cultures were then transferred onto fresh PDA plates without antibiotics, incubated until plates were confluent, and then stored at 4 °C. For long-term storage, cultures were maintained as agar plugs in sterile distilled water as described previously (Boesewinkel, 1976).

### Species identification

To confirm species identity, internal transcribed spacer (ITS) sequencing was carried out. DNA was extracted from freeze-dried fungal mycelium using the protocol from Ward *et al.* (2005) (modified from Fraaije *et al.*, 1999). PCR was done to amplify the ITS regions using primers ITS5 (GGAAGTAAAAGTCGTAACAAGG) and ITS4 (TCCTCCGCTTATTGATATGC) (White *et al.*, 1990). Each 20 µl reaction contained 10 µl of *Taq* polymerase (REDTaq^®^ ReadyMix™ PCR Reaction Mix, Sigma-Aldrich), 1 μl of each primer (10 μM), 6 μl of sterile distilled H_2_O, and 2 μl of template DNA (100 ng µl^–1^). PCR conditions were: 95 °C 5 min, 30 cycles of 95 °C 30 s, 55 °C 1 min, 72 °C 1 min, and extension at 72 °C for 10 min. PCR products were purified using a Qiagen QIAquick PCR Purification Kit, sequenced, and identification was confirmed using the BLAST tool and searching the NCBI database.

### Seedling pot bioassay with addition of artificial inoculum

A seedling pot bioassay was designed to evaluate the susceptibility of cereal genotypes to one representative isolate of *G. hyphopodioides* (N.14.13) and the unnamed Magnaporthaceae species (S.09.13) from the culture collection. A range of cereal genotypes were evaluated including those used as controls in the take-all seedling pot bioassay: oats (cv. Gerald, immune to take-all), rye (cv. Carotop, highly resistant to take-all), triticale (cv. Trilogie, moderately resistant to take-all), and hexaploid wheat (cv. Hereward, highly susceptible to take-all) (McMillan *et al.*, 2014). Additional hexaploid wheat genotypes were the spring wheat commercial cultivar Paragon and the Watkins landrace line 1190777; Paragon is susceptible to take-all whilst Watkins line 1190777 is partially resistant to take-all (VM, unpublished data), and the *Triticum monococcum* genotypes MDR037 (susceptible to take-all) and MDR046 (moderately resistant to take-all) (McMillan *et al.*, 2014). Hereward was also used as a negative control in both pot bioassays with non-inoculated PDA.

A randomized block design was calculated in GenStat (VSNI, Hemel Hempstead, UK) (Payne *et al.*, 2009) and included three inoculated replicates for each treatment. Soil (type: typical Batcombe) was collected in September 2013 from Great Harpenden I field (after oats) on the Rothamsted Farm, crumbled, mixed and then stored at room temperature before use in the seedling pot bioassay. Plastic drinking cups (7.5 cm diameter × 11 cm height, drilled with four drainage holes, 3 mm diameter) were filled with a 50 cm^3^ layer of damp coarse sand and then a 150 g layer of soil. PDA plate inoculum was prepared by macerating 1/6th of a confluent PDA plate of either *G. hyphopodioides* or the Magnaporthaceae sp. with soil, equating to a ~25 g layer. The negative control pots were prepared by macerating 1/6th of a non-colonized PDA plate with soil. A further 50 g of prepared soil was added on top. The soil was lightly watered and 10 seeds of each cultivar were placed on the soil surface. Seeds were covered with a ~2 cm layer of horticultural grit and the pots were placed in a controlled-environment room (16 h day, 15 °C day/10°C night, twice weekly watering) for 5 weeks. After 5 weeks, the roots were washed free of soil and immersed in a white dish to examine the roots visually for colonization by looking for the presence of subepidermal vesicles. The total number of plants and roots and the number of colonized plants and roots were recorded to calculate the percentages of plants and roots infected.

### Field trials

Two field trials, to evaluate the ability of elite UK winter wheat cultivars to support natural populations of the *G. hyphopodioides* fungus under a first wheat crop, were established in autumn 2014 and 2015. The two small plot field trials were established in two different parts of the same field, known to have underlying natural populations of *G. hyphopodioides*, on the Rothamsted Farm (Hertfordshire, UK) (Supplementary Table S2). The soil is flinty clay loam soil of the typical Batcombe soil series. The experimental field trials consisted of randomized block designs of five replicates of 40 elite wheat cultivars. The elite wheat cultivars consisted of 36 winter wheat cultivars on the Agriculture and Horticulture Development Board (AHDB) 2013/2014 Recommended List (RL) and two winter wheat cultivars (Evolution and Zulu) on the AHDB 2014/2015 RL. In addition two control cultivars were included, the spring wheat cultivar Cadenza and the winter wheat cultivar Hereward, both with known take-all inoculum-building phenotypes (low and high, respectively) (McMillan *et al.*, 2011).

The two field trials were grown as first wheat crops, after a 1 year break crop of winter oilseed rape (2014), the second after winter oilseed rape and then spring oats (2015). Fertilizers, pesticides, and growth regulators were applied according to the standard practice of the Rothamsted Farm (Supplementary Table S3).

### Soil core bioassay to gauge the amount of fungal inoculum under the first wheat crop

Post-harvest soil cores were taken from each plot to set up a soil core bioassay (McMillan *et al.*, 2011) to gauge the infectivity of *G. hyphopodioides* fungal inoculum in the soil under the different elite wheat cultivars. The method involved baiting soil cores with wheat seedlings, and fungal colonization was then assessed visually after 5 weeks growth in the controlled-environment room. The baited wheat seedlings effectively represent a subsequent second wheat crop. Six soil cores (5.5 cm diameter by 10 cm depth) were taken post-harvest in a zig–zag transect from different rows across individual plots using a soil auger. Three of the soil cores were watered and 10 seeds of the winter wheat cultivar Hereward (RAGT, Cambridge, UK) were placed on the surface of each of the cores to gauge the amount of infective fungal inoculum after growth of current commercial cultivars. Ten seeds of the field plot cultivar were placed on the surface of each of the three remaining soil cores to test for the possibility of wheat genotype–fungal colonization interactions. After 5 weeks growth, the plant roots were washed free of soil and immersed in water in a white dish to examine the roots visually for *G. hyphopodioides* colonization. Any *G. tritici* lesions were also recorded to identify whether take-all fungal inoculum could build up in a field with underlying *G. hyphopodioides* populations. The percentage of colonized roots was calculated for the two baiting methods and to gauge the amounts of *G. hyphopodioides* or *G. tritici* inoculum that were supported under each wheat cultivar for the Hereward baiting. Cultures were isolated from colonized root tissue from soil core bioassay seedling plants, as detailed in the previous pot bioassay fungal isolation methodology, to confirm visual assessments that *G. hyphopodioides* was the species present.

### Statistical analyses

The colonization percentages were always transformed using the logit transformation to ensure equal variance. The transformed data from the pot bioassay with different cereal genotypes was then statistically analysed using ANOVA in GenStat (VSN International Ltd).

For the field data, a residual maximum likelihood (REML) variance components analysis was used to incorporate the sub-blocking structure within the field trials, and autoregressive models were used when required for spatial adjustment of the field trials to account for the degree of patchiness of fungal inoculum in both the *y*-axis and the *x*-axis across the trial sites. Yield data from the two field trials were also statistically analysed using a REML variance components analysis. A combined REML variance components analysis was then used to pool and analyse data from across the two field seasons together. The *P*-value threshold was set at ≤0.05 for all tests.

### Microscopy analysis

A LEICA M205 FA stereomicroscope and associated LAS-AF6000 software (Leica Microsystems Ltd, UK) were used for all microscopic visualization and image capture of fungi in the colonized roots. Seedling roots were submerged in water in a Petri dish and visualized under the stereomicroscope. Scale bars were generated by the LAS-AF6000 software.

### Phylogenetic analysis

The 47 *G. hyphopodioides* ITS5–ITS4 rDNA regions, from the pot bioassay and two field trials, were compared with ITS rDNA regions of the top three BLAST hits from the NCBI database for all isolates as well as a subset of *G. graminis*, *G. hyphopodioides*, *G. tritici*, and the unnamed Magnaporthaceae sp. isolates (Hernández-Restrepo *et al.*, 2016). All ITS5–ITS4 rDNA regions for all species were aligned in the software package Geneious (Biomatters Ltd v8.1.3), and a 498 bp region was extracted. A phylogenetic tree was constructed on the 498 bp region using the genetic distance model of Tamura and Nei, the tree build method of Neighbor–Joining with 1000 bootstrap replicates, and a support threshold set at 75% in Geneious. The phylogenetic tree was rooted with the *Pyricularia grisea* strains BR0029 and CR0024. Accession numbers for sequences obtained from the NCBI database can be found in Supplementary Table S4.

## Results

### Fungal isolations and phylogenetic analysis

An isolate collection was gathered from soil taken post-harvest from four commercial cereal crops harvested in 2013. The field sites chosen had previously shown some suppression of take-all disease in field experiments carried out between 2009 and 2012 (Supplementary Fig. S1). *Gaeumannomyces hyphopodioides*, the unnamed Magnaporthaceae sp., and other closely related fungal root colonizers in the same family produce subepidermal vesicles within the root cortex (Deacon, 1974). All of the sampled field sites showed this root colonization phenotype with between 18% and 82% of cores displaying characteristic symptoms for each field (Supplementary Table S1). In total, nine isolates that had formed subepidermal vesicles in the correct size range were recovered from the wheat seedlings for further analysis from three sites (Table 1). DNA sequences for the ITS5–ITS4 region were obtained and eight isolates from the collection (excluding isolate S.09.13) showed 99–100% species identity with *G. hyphopodioides* (NCBI Taxonomy ID: 1940676) strain CPC 26267, *G. hyphopodioides* strain CPC 26249, and *G. hyphopodioides* strain CPC 26248 (Hernández-Restrepo *et al.*, 2016), the top three hits for all isolates from the NCBI database (Table 1; Supplementary Table S4). The ITS5–ITS4 rDNA sequence for the S.09.13 strain from the initial isolate collection showed 99% species identity with the unnamed Magnaporthaceae sp., an uncultured *Phialophora* species isolated in 2009 (NCBI taxonomy ID: 268601) (Moll *et al.*, 2016), Magnaporthaceae sp. (NCBI taxonomy ID: 1940802) strain CPC 26284 (Hernández-Restrepo *et al.*, 2016), and Magnaporthaceae sp. isolate 437 (Ulrich *et al.*, 2000) (Table 1; Supplementary Table S4). Interestingly, both *G. hyphopodioides* and the unnamed Magnaporthaceae sp. were isolated from the same field in the case of Summerdells I, whereas only *G. hyphopodioides* was recovered from the other two fields (New Zealand and Pastures).

**Table 1.** Fungal isolate identity in the initial collection from the field season year 2013 and isolates obtained from the two experimental field trials in the field season years 2015 and 2016

Isolate code	Original field host and cultivar	Soil bioassay host and cultivar	RRes field name	Fungal identity
Initial isolate collection^*a*^				
N.14.13^*b*,*c*^	*Hordeum vulgare*, Tipple	*T. aestivum*, Hereward	New Zealand	*G. hyphopodioides*
N.20.13	*Hordeum vulgare*, Tipple	*T. aestivum*, Hereward	New Zealand	*G. hyphopodioides*
P.03.13	*T. aestivum*, Conqueror	*T. aestivum*, Hereward	Pastures	*G. hyphopodioides*
P.05.13	*T. aestivum*, Conqueror	*T. aestivum*, Hereward	Pastures	*G. hyphopodioides*
P.06.13	*T. aestivum*, Conqueror	*T. aestivum*, Hereward	Pastures	*G. hyphopodioides*
P.09.13	*T. aestivum*, Conqueror	*T. aestivum*, Hereward	Pastures	*G. hyphopodioides*
P.10.13	*T. aestivum*, Conqueror	*T. aestivum*, Hereward	Pastures	*G. hyphopodioides*
S.03.13	*T. aestivum*, Conqueror	*T. aestivum*, Hereward	Summerdells I	*G. hyphopodioides*
S.09.13^*d*^	*T. aestivum*, Conqueror	*T. aestivum*, Hereward	Summerdells I	Magnaporthaceae sp.
2015/R/WW/1516 field trial				
NZ.16.1A^*e*^.15	*T. aestivum*, Zulu	*T. aestivum*, Hereward	New Zealand	*G. hyphopodioides*
NZ.24.2A.15	*T. aestivum*, KWS Kielder	*T. aestivum*, KWS Kielder	New Zealand	*G. hyphopodioides*
NZ.112.1A.15	*T. aestivum*, KWS Target	*T. aestivum*, Hereward	New Zealand	*G. hyphopodioides*
NZ.136.1A.15	*T. aestivum*, Tuxedo	*T. aestivum*, Hereward	New Zealand	*G. hyphopodioides*
NZ.141.2A.15	*T. aestivum*, Duxford	*T. aestivum*, Duxford	New Zealand	*G. hyphopodioides*
NZ.155.1A.15	*T. aestivum*, Revelation	*T. aestivum*, Hereward	New Zealand	*G. hyphopodioides*
NZ.160.1A.15	*T. aestivum*, KWS Sterling	*T. aestivum*, Hereward	New Zealand	*G. hyphopodioides*
NZ.173.2A.15	*T. aestivum*, Solstice	*T. aestivum*, Solstice	New Zealand	*G. hyphopodioides*
NZ.8.1B.16	*T. aestivum*, Delphi	*T. aestivum*, Hereward	New Zealand	*G. hyphopodioides*
NZ.12.2B.16	*T. aestivum*, Solstice	*T. aestivum*, Solstice	New Zealand	*G. hyphopodioides*
NZ.110.2B.16	*T. aestivum*, Cordiale	*T. aestivum*, Cordiale	New Zealand	*G. hyphopodioides*
NZ.43.1C.16	*T. aestivum*, Relay	*T. aestivum*, Hereward	New Zealand	*G. hyphopodioides*
NZ.93.2C.16	*T. aestivum*, JB Diego	*T. aestivum*, JB Diego	New Zealand	*G. hyphopodioides*
NZ.103.2C.16	*T. aestivum*, Solstice	*T. aestivum*, Solstice	New Zealand	*G. hyphopodioides*
NZ.136.1C.2.16	*T. aestivum*, Tuxedo	*T. aestivum*, Hereward	New Zealand	*G. hyphopodioides*
NZ.138.1C.16	*T. aestivum*, Zulu	*T. aestivum*, Hereward	New Zealand	*G. hyphopodioides*
NZ.176.1C.16	*T. aestivum*, Evolution	*T. aestivum*, Hereward	New Zealand	*G. hyphopodioides*
NZ.183.1C.16	*T. aestivum*, Invicta	*T. aestivum*, Hereward	New Zealand	*G. hyphopodioides*
NZ.184.1C.16	*T. aestivum*, Monterey	*T. aestivum*, Hereward	New Zealand	*G. hyphopodioides*
2016/R/WW/1620 field trial				
NZ.3.2A.17	*T. aestivum*, Scout	*T. aestivum*, Scout	New Zealand	*G. hyphopodioides*
NZ.143.1A.17	*T. aestivum*, KWS Croft	*T. aestivum*, Hereward	New Zealand	*G. hyphopodioides*
NZ.198.1A.17	*T. aestivum*, Invicta	*T. aestivum*, Hereward	New Zealand	*G. hyphopodioides*
NZ.38.1B.17	*T. aestivum*, KWS Sterling	*T. aestivum*, Hereward	New Zealand	*G. hyphopodioides*
NZ.46.1B.17	*T. aestivum*, Relay	*T. aestivum*, Hereward	New Zealand	*G. hyphopodioides*
NZ.86.2B.17	*T. aestivum*, Einstein	*T. aestivum*, Einstein	New Zealand	*G. hyphopodioides*
NZ.109.1B.17	*T. aestivum*, Grafton	*T. aestivum*, Hereward	New Zealand	*G. hyphopodioides*
NZ.114.2B.17	*T. aestivum*, KWS Gator	*T. aestivum*, KWS Gator	New Zealand	*G. hyphopodioides*
NZ.148.1B.17	*T. aestivum*, Relay	*T. aestivum*, Hereward	New Zealand	*G. hyphopodioides*
NZ.164.1B.17	*T. aestivum*, Monterey	*T. aestivum*, Hereward	New Zealand	*G. hyphopodioides*
NZ.185.2B.17	*T. aestivum*, Cordiale	*T. aestivum*, Cordiale	New Zealand	*G. hyphopodioides*
NZ.23.1C.17	*T. aestivum*, Viscount	*T. aestivum*, Hereward	New Zealand	*G. hyphopodioides*
NZ.41.2C.17	*T. aestivum*, KWS Gator	*T. aestivum*, KWS Gator	New Zealand	*G. hyphopodioides*
NZ.104.1C.17	*T. aestivum*, KWS Sterling	*T. aestivum*, Hereward	New Zealand	*G. hyphopodioides*
NZ.115.1C.17	*T. aestivum*, KWS Target	*T. aestivum*, Hereward	New Zealand	*G. hyphopodioides*
NZ.129.2C.17	*T. aestivum*, Scout	*T. aestivum*, Scout	New Zealand	*G. hyphopodioides*
NZ.135.2C.17	*T. aestivum*, Solstice	*T. aestivum*, Solstice	New Zealand	*G. hyphopodioides*
NZ.155.2C.17	*T. aestivum*, Cordiale	*T. aestivum*, Cordiale	New Zealand	*G. hyphopodioides*
NZ.160.2C.17	*T. aestivum*, Cadenza	*T. aestivum*, Cadenza	New Zealand	*G. hyphopodioides*

^*a*^ No isolates were recovered from Great Knott III RRes field on the Rothamsted Farm.

^b^ Year of isolation is represented by the last two digits of the isolate ID, e.g. N.14.13 was isolated in 2013.

^*c*^ *Gaeumannomyces hyphopodioides* isolate N.14.13 was used in the pot bioassay to screen the susceptibility of different cereal species and genotypes.

^*d*^ Magnaporthaceae sp. isolate S.09.13 was used in the pot bioassay to screen the susceptibility of different cereal species and genotypes.

^*e*^ The post-harvest soil core bioassays from the two field trials were split into three groups to give one pot replicate per plot per group when assessing the roots for *G. hyphopodioides* colonization, and therefore the codes A, B, and C represent isolates from each of the three groups.

Further isolates were obtained from colonized root tissue of the soil core bioassay plants from the two experimental field trials in New Zealand field to confirm the presence of *Gaeumannomyces* species. The ITS5–ITS4 rDNA sequences for all 19 isolates from the 2015 field trial and all 19 isolates from the 2016 field trial also showed 99–100% species identity with the three *G. hyphopodioides* strains (CPC 26267, CPC 26249, and CPC 26248) (Hernández-Restrepo *et al.*, 2016), and were found to be the top three hits for all isolates from the NCBI database (Table 1). The ITS5–ITS4 region was highly conserved across all *G. hyphopodioides* isolates recovered in 2013, 2015, and 2016, with only one single nucleotide polymorphism (SNP) across all 47 isolates.

**Fig. 1.**
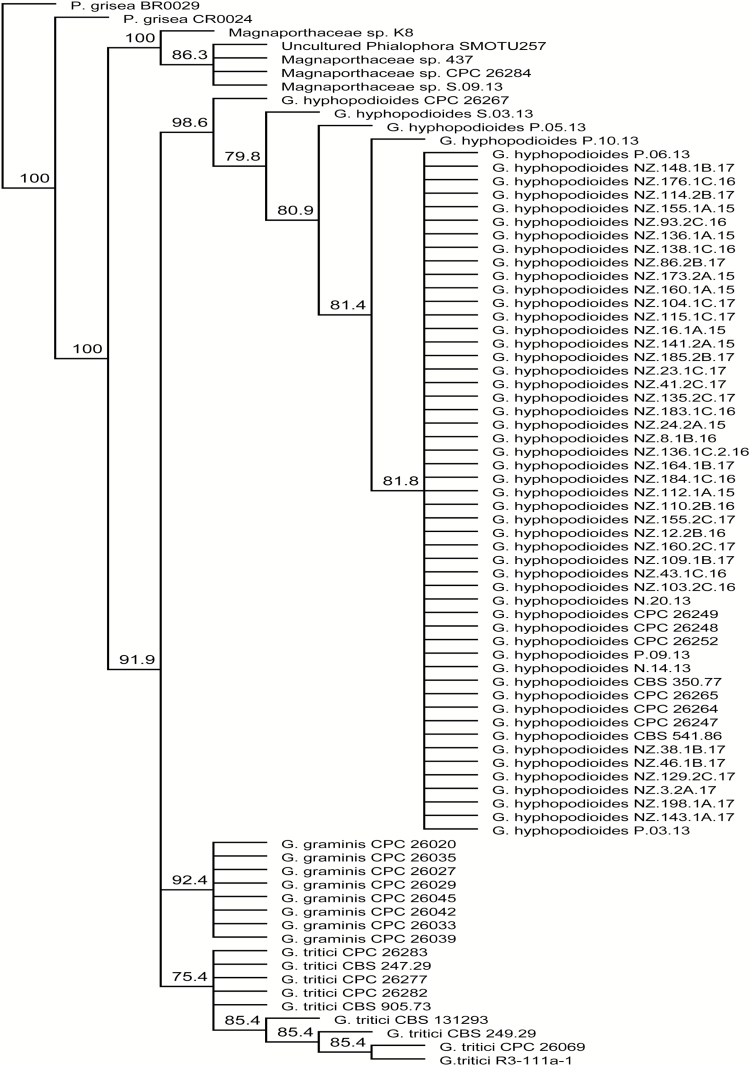
Phylogenetic tree of the ITS5–ITS4 rDNA regions of isolates from the initial isolate collection and *Gaeumannomyces hyphopodioides* isolates from the two experimental field trials, along with sequences obtained from the NCBI database of species within Magnaporthaceae. The genetic distance model of Tamura and Nei was used and a tree build method of Neighbor–Joining was performed with 100 bootstraps. A 75% support threshold was used.

**Fig. 2.**
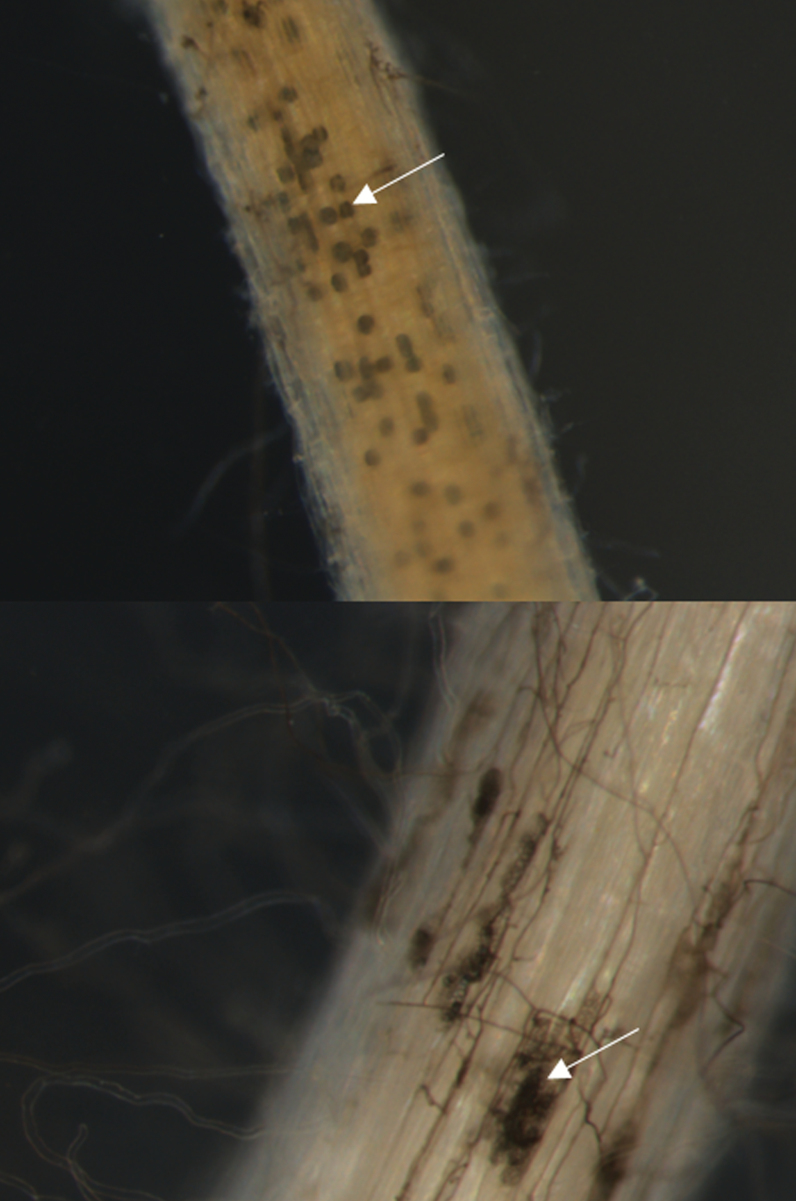
*Gaeumannomyces hyphopodioides*-colonized wheat (cultivar Hereward, isolate P.10.13) seedling root (a). The white arrow indicates the colonization phenotype of large, single subepidermal vesicles, magnification ×67. Unnamed Magnaporthaceae sp.-colonized wheat (cultivar Hereward, isolate S.09.13) seedling root (b). White arrows indicate the colonization phenotype of small and clustered subepidermal vesicles magnification ×92.3.

A phylogenetic analysis was constructed to identify the genetic relationship between isolates within the initial isolate collection (*n*=9) and the isolates obtained from the two experimental field trials (*n*=38), as well as the relationship of these isolates to 32 reference isolates downloaded from the NCBI database (Supplementary Table S4). The Magnaporthaceae sp. isolate S.09.13, recovered from the initial 2013 isolate collection, clusters with all the unnamed Magnaporthaceae sp. isolates in the NCBI database. The Magnaporthaceae sp. form a separate clade from both *Gaeumannomyces* species (Fig. 1). The *G. hyphopodioides* isolates recovered in 2013, 2015, or 2016 and reference NCBI isolates all cluster together, separate from the *G. graminis* and *G. tritici* isolates obtained from the NCBI (Fig. 1). Therefore, these data confirm that the complete isolate collection contains two distinct species within the Magnaporthaceae and these form two distinct clades, confirming the reassessed taxonomy of the group by Hernández-Restrepo *et al.* (2016). Colonized seedling roots, from the initial isolate collection, were examined under the light microscope and photographs were captured to illustrate the two colonization phenotypes identified (Fig. 2). The characteristic large, single subepidermal vesicles were found for *G. hyphopodioides*-colonized roots (Deacon 1974) (Fig. 2a) (isolate P.10.13; Table 1) and small clusters of smaller subepidermal vesicles were found for seedling roots colonized by the unnamed Magnaporthaceae sp. (Ulrich *et al.*, 2000) (Fig. 2b) (isolate S.09.13; Table 1).

### Cereal genotype root colonization in seedling pot bioassay

A seedling pot bioassay with addition of an artificial inoculum was devised to evaluate the ability of the two fungal species within the Magnaporthaceae isolate collection to colonize the roots of selected cereal species and wheat genotypes. Two experimental pot bioassays were carried out and a significant interaction was identified in the percentage of colonized roots between the two fungal species across the eight cereal genotypes (*P*<0.001) (Table 2). A ~50% level of colonization of the roots for the wheat cultivar Hereward was reached, providing a benchmark to allow good discrimination. There was a statistically significant difference in the main effect of percentage of roots colonized by the two fungal species in the second pot bioassay [ANOVA: *P*<0.001, df=1, standard error of the difference (SED)=0.160] but not for the first pot bioassay (ANOVA: *P*=0.168, df=1, SED=0.152). However, particularly noticeable was the low level of fungal colonization of oat roots for both species. A high level of fungal colonization was observed across the diploid wheat (*T. monococcum*), hexaploid wheat, and triticale cultivars, whereas in a take-all bioassay triticale is moderately resistant (McMillan *et al.*, 2011). For rye, there was a low level of colonization for the unnamed Magnaporthaceae sp. but higher levels for *G. hyphopodioides*. Overall, the percentage of roots colonized by the unnamed Magnaporthaceae sp. was statistically significantly higher than the percentage of roots colonized by *G. hyphopodioides* for all cereal genotypes, except rye (Table 2) where the reverse outcome was clearly evident. Representative colonization phenotypes for both species are shown in Fig. 2.

**Table 2.** Ability of *Gaeumannomyces hyphopodioides* and Magnaporthaceae sp. to colonize cereal roots in a potato dextrose agar (PDA) inoculated seedling pot bioassay in soil

Fungal species	Cereal genotype and cultivar	Logit percentage of colonized roots (back-transformed means)			
		First pot bioassay		Second pot bioassay	
*Gaeumannomyces hyphopodioides*	Oats, Gerald	–4.05	(1.23)	–3.68	(1.99)
	Rye, Carotop	–0.88	(29.17)	–1.11	(24.48)
	Triticale, Trilogie	–0.26	(43.56)	–1.42	(19.22)
	*T. aestivum*, Hereward	–0.41	(39.82)	–0.37	(40.79)
	*T. aestivum*, Hereward -^a^	–2.12	(10.29)	–5.30	(0)
	*T. aestivum*, Paragon	–0.83	(30.14)	–1.03	(26.68)
	*T. aestivum*, Watkins 1190777	–0.37	(40.75)	–0.10	(48.13)
	*T. monococcum*, MDR037	–0.68	(33.42)	–0.07	(33.30)
	*T. monococcum*, MDR046	–0.23	(44.15)	–0.69	(26.05)
Unnamed *Magnaporthaceae species*	Oats, Gerald	–2.72	(5.75)	–2.40	(7.89)
	Rye, Carotop	–2.99	(4.33)	–2.63	(6.27)
	Triticale, Trilogie	–0.01	(49.76)	–0.06	(48.61)
	*T. aestivum*, Hereward	0.03	(50.69)	0.41	(60.11)
	*T. aestivum*, Hereward -^*a*^	–1.85	(13.22)	–4.37	(0.76)
	*T. aestivum*, Paragon	–0.65	(34.16)	0.24	(57.35)
	*T. aestivum*, Watkins 1190777	0.09	(52.34)	0.29	(66.20)
	*T. monococcum*, MDR037	0.33	(58.32)	0.67	(53.28)
	*T. monococcum*, MDR046	–0.15	(46.35)	0.13	(56.13)
	df	8		8	
	SED (logit scale)	0.455		0.481	
	F probability	<0.001		0.005	

^*a*^ Hereward - =Hereward negative control with non-colonized PDA. Microscopic analysis revealed very small clustered subepidermal vesicles and the species is thought to be either the unnamed Magnaporthaceae sp. or *Slopeiomyces cylindrosporus* (NCBI Taxonomy ID: 1577607) (Klaubauf *et al.*, 2014); unfortunately this isolate was not recovered.

### Colonization of UK winter wheat cultivars under field conditions

**Fig. 3.**
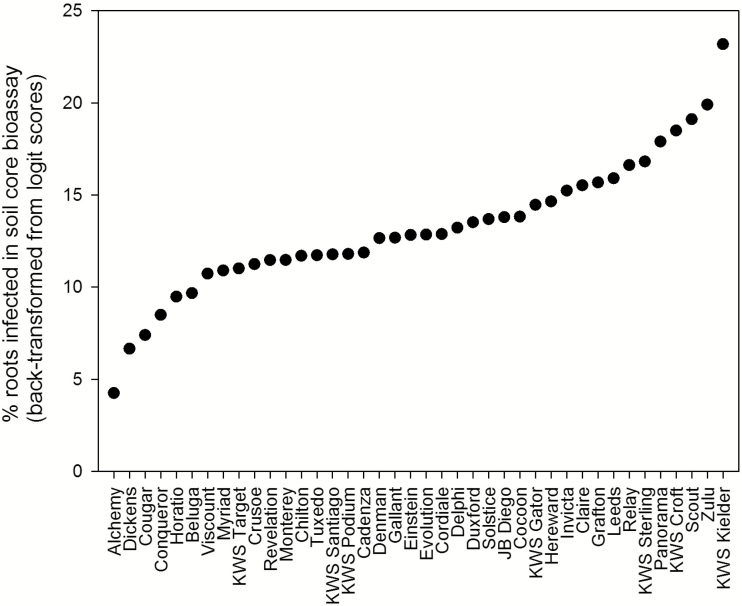
Percentage of roots colonized with *Gaeumannomyces hyphopodioides* (back-transformed means of the logits) when baited with the winter wheat cultivar Hereward in the soil core bioassay. Combined analysis of data pooled across the two years (χ^2^ probability <0.001, SED (logit scale)=0.171, Wald statistic=637.76). See Supplementary Table S5 for data on logit scale.

The third aim of the study was to explore whether there were any differences in the ability of current commercial UK winter wheat cultivars to support natural populations of *G. hyphopodioides* in the field in a first wheat situation, measured by their colonization of a subsequent crop in the seedling soil core bioassay. Soil cores taken from the two field trials and subsequently assessed in the seedling soil core bioassay, baited with Hereward, revealed that there were differences between elite wheat cultivars (Fig. 3; Supplementary Tables S5, S6). The overall level of *G. hyphopodioides* inoculum, measured by the percentage of root colonization of Hereward, differed across the two years. The field trial grand mean in 2016 (7.55%) was almost double the grand mean in 2015 (3.82%) (Supplementary Table S5). Correlation between the two years was low (*r*_s_= –0.04, *P*=0.798), with many cultivars showing contrasting results; for example, Hereward seedlings sown after Gallant had 5% of roots colonized in the soil core bioassay in the 2015 field trial and 17% of roots colonized in the 2016 field trial. However, there was a subset of cultivars which were consistently low in supporting *G. hyphopodioides* inoculum in both years (e.g. Alchemy and Dickens), as well as cultivars consistently supporting higher levels of inoculum in the two trial years (Zulu, KWS Croft, KWS Kielder, and KWS Sterling) (Supplementary Table S5). When data were pooled from both years in a combined REML variance components analysis, there was an overall significant effect of cultivar, revealing that Alchemy was the lowest supporter of *G. hyphopodioides* inoculum, whereas KWS Kielder supported the highest levels of *G. hyphopodioides* inoculum, 18% higher than for Alchemy (Fig. 3). Eleven cultivars supported higher levels of *G. hyphopodioides* inoculum than the control cultivar of Hereward (Fig. 3).

**Fig. 4.**
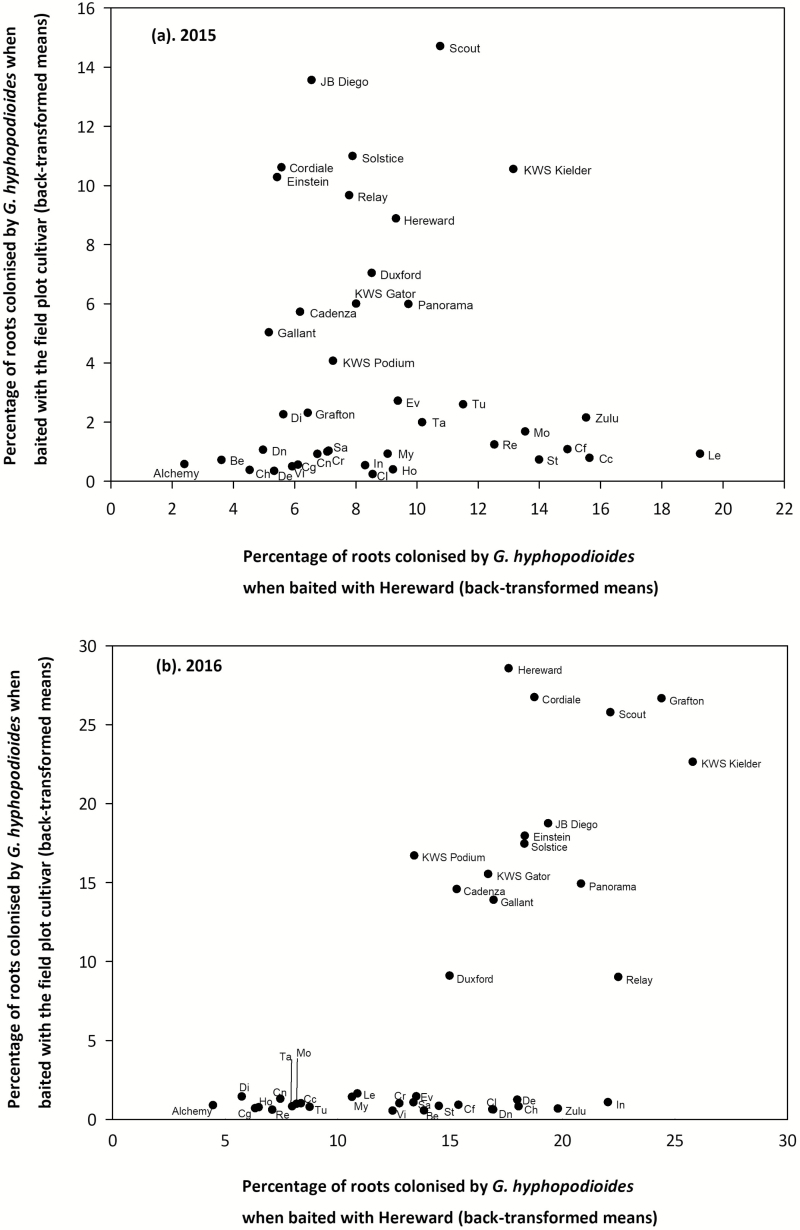
Correlation between percentage of roots colonized with *Gaeumannomyces hyphopodioides* (back-transformed means of the logits) when baited with the field plot cultivar or Hereward in the soil core bioassay in 2015 (a) [*P*<0.001, SED (logit scale)=0.231, *F*-statistic=5.58] and 2016 (b) [*P*<0.001, SED (logit scale)=0.194, *F*-statistic=13.50]. Be, Beluga; Cc, Cocoon; Cf, KWS Croft; Cg, Cougar; Ch, Chilton; Cl, Claire; Cn, Conqueror; Cr, Crusoe; De, Delphi; Di, Dickens; Dn, Denman; Ev, Evolution; Ho, Horatio; In, Invicta; Le, Leeds; Mo, Monterey; My, Myriad; Re, Revelation; Sa, KWS Santiago; St, KWS Sterling; Ta, KWS Target; Tu, Tuxedo and Vi, Viscount. Very low root colonization, <5%; low root colonization, 5*–*10%, medium root colonization, >10%.

The fourth aim of this study was to establish whether there was any interaction between second wheat cultivar choice, used as the baiting cultivar in the soil core bioassay, and their subsequent level of root colonization by *G. hyphopodioides*. To address this, half of the soil cores were baited back on themselves with the same cultivar grown in the field trial and compared with the cores previously baited with the highly take-all-susceptible cultivar Hereward. Most winter wheat cultivars were found to be poorly colonized by *G. hyphopodioides* when baited with the same field plot cultivar (25/40 cultivars) in both experiments (<5% of roots infected; Fig. 4). However, a subset of cultivars, including cultivars Einstein, Solstice, JB Diego, KWS Kielder, Scout, and Cordiale, consistently had higher levels (>10% of roots) of *G. hyphopodioides* root colonization in both years (Fig. 4; Supplementary Table S5). A strong correlation (*r*_s_=0.765, *P*<0.001) between the two years in the level of root colonization by *G. hyphopodioides* was found, in contrast to the low correlation found when baited with Hereward in aim three.

A significant interaction was found for the second wheat cultivar choice across the 40 cultivars (2015, *P*<0.001; 2016, *P*<0.001), with a trend for a higher percentage of roots colonized with *G. hyphopodioides* when baited with Hereward for most elite winter wheat cultivars (17 cultivars had ≥10% of roots colonized with Hereward across one or both field trials), with only eight cultivars giving a higher percentage of colonized roots when baited with the field plot compared with when baited with Hereward (Supplementary Table S5). The 25 winter wheat cultivars that were found to support low colonization of *G. hyphopodioides* when the second wheat cultivar was the field plot cultivar were found to support higher levels of root colonization when the second wheat cultivar was Hereward, except for Alchemy (Supplementary Table S5). Inconsistencies in the level of root colonization between the two baiting methods is highly evident for cultivars Zulu, Leeds, and KWS Croft (Supplementary Table S5). In contrast, there were no cultivars that had a very low percentage of root colonization by *G. hyphopodioides* (<5%) when baited with Hereward in the soil core bioassay, as well as having a moderate percentage of roots colonized when baited with the field plot cultivar (Fig. 4). A pooled cross-season REML variance components analysis across the 40 cultivars revealed that nine cultivars supported medium levels of *G. hyphopodioides* root colonization (>10% of roots colonized), regardless of second wheat cultivar choice (Supplementary Table S6).

Although the field trial site has natural underlying populations of *G. hyphopodioides*, the soil core bioassay plants were also assessed for any visible take-all infection. As expected, there was a negligible amount of take-all across the field site for both field trial years, with <2.1% of roots infected with take-all across all cultivars (Supplementary Tables S7, S8).

The plot yields were taken from both experimental field trials and there were significant effects of cultivar on plot yields for both field trial years (2015, *P*<0.001; 2016, *P*<0.001) (Supplementary Table S9). No correlation was found between the plot yields and the percentage of roots colonized with *G. hyphopodioides* when baited with Hereward, in the soil core bioassay, for the 2015 field trial (*r*_s_=0.102, *P*=0.133, *n*=40), but a weak negative correlation was found for the 2016 field trial (*r*_s_= –0.228, *P*=0.039, *n*=40). No correlations were found between the plot yields and the percentage of roots colonized with *G. hyphopodioides* when baited with the field plot cultivar, in the soil core bioassay, for either field trial year (2015, *r*_s_= –0.100, *P*=0.134, *n*=40; 2016, *r*_s_= –0.099, *P*=0.136, *n*=40).

## Discussion

In this study, a new UK arable soil-derived collection of *G. hyphopodioides* and Magnaporthaceae sp. isolates was obtained over three cropping seasons and characterized with existing information from the recent taxonomical reclassification of the Magnaporthaceae family by Hernández-Restrepo *et al.* (2016). A seedling pot bioassay with artificial inoculum addition then revealed that there were differences in the susceptibility of five cereal species at the seedling stage to the two fungal species. The winter wheat cultivar Hereward was found to be highly susceptible in the artificial pot bioassay to both fungal species, and was subsequently chosen to be used as the baiting cultivar in the seedling soil core bioassay to test the difference between cultivars in their ability to support *G. hyphopodioides* inoculum under field trial conditions. There was some evidence of a difference between cultivars in their ability to support *G*. *hyphopodioides* inoculum under the first wheat crop (gauged using Hereward as the baiting cultivar), although this was not very consistent across the two trial years, indicating a strong genotype×environment component. In contrast, there were more consistent differences between cultivars in the ability of *G. hyphopodioides* to colonize seedlings in the soil core bioassay, when baited with the field plot cultivar. We discovered that by changing the hexaploid wheat cultivar used as the bait in the soil core bioassay, the level of *G. hyphopodioides* root colonization was often altered. Collectively, these new results provide valuable information on how beneficial soil-dwelling fungi can be encouraged to proliferate in arable soils to benefit wheat root health and hence grain production.

The first aim of the study was to gather an isolate collection from arable fields on an experimental farm in south-east England. There was a higher recovery of isolates of the *G. hyphopodioides* species compared with the unnamed Magnaporthaceae sp. The two species were only recovered together from one field, whereas in two other fields only *G. hyphopodioides* was recovered. No isolates were recovered from the fourth sampled field (Great Knott III) where beneficial Magnaporthaceae sp. had previously been visually identified ~8 years previously (VM, personal communication). No isolates of *Slopeiomyces cylindrosporus* (Klaubauf *et al.*, 2014) (anamorph: *Phialophora graminicola*; Walker, 1980), previously isolated and studied in Rothamsted field trials (Ward and Gray, 1992; Bryan *et al.*, 1995), were isolated. Collectively these results indicate that the populations of these soil-dwelling beneficial fungal species are not static.

The 47 *G. hyphopodioides* isolates gathered from the various sites/trials across the Rothamsted Farm were found to be highly conserved across the ITS region. This isolate collection is an important resource for future studies. Experiments are already underway to sequence and fully assemble the genomes of different Magnaporthaceae species within the collection, and comparative studies with *G. tritici* should permit an improved understanding of the key differences between these closely related soil-dwelling beneficial and pathogenic species. The isolate collection could also be used to design a species-specific diagnostic assay to allow the identification of the different beneficial fungi present in arable fields.

The isolate collection was further used in the current study to establish a seedling pot bioassay under controlled environmental conditions, with the aim of exploring the root colonization of different cereal species by non-pathogenic soil-borne Magnaporthaceae species. Triticale had a high level of colonization for both fungi, whereas triticale is moderately resistant to the take-all fungus (McMillan *et al.*, 2014). The remaining cereal genotypes, including the ancestral wheat relative *T. monococcum* (A^m^ genome), the hexaploid wheat landrace Watkins 1190777, and the semi-modern elite spring and winter wheat genotypes appeared to be equivalent in their level of fungal colonization at the seedling stage. This result suggests that fungal colonization by beneficial *Gaeumannomyces* species has not been significantly altered by intensive wheat breeding activities. Rye had a low level of root colonization by the unnamed Magnaporthaceae sp., suggesting that rye could be activating a similar defence mechanism against the fungus to that observed with take-all (Rothrock 1988). The naïve soil used to establish all the pot bioassays was not sterilized, which explains why very low levels of visible subepidermal vesicles could be found on the roots of the non-inoculated Hereward control roots.

The third aim of this study was to investigate whether there were any differences in the ability of current commercial UK winter wheat cultivars to support natural populations of *G. hyphopodioides* in the field in a first wheat situation. The two years of Hereward baiting data revealed that there were differences in the ability of the elite wheat cultivars to support *G. hyphopodioides* inoculum under a first wheat crop. However, there were inconsistencies in the level of root colonization for cultivars between the two years, highlighting a genotype×environment interaction. The higher level of *G. hyphopodioides* root colonization in the 2016 field trial suggests that the 2015–2016 season was more environmentally conducive to supporting natural populations of *G. hyphopodioides*. Weather conditions in 2015 consisted of a wet spring and summer compared with a drier spring and summer in 2016 (Supplementary Table S10). This contrasts with take-all disease which is generally favoured by warmer winters and wet springs/summers. Alternatively, differences in field site location may account for differences in levels of *G. hyphopodioides* inoculum between the two years.

The wheat genotype–*G. hyphopodioides* interaction detected in aim three of this study complements an earlier study that had identified consistent differences in the ability of wheat cultivars to build up take-all (*G. tritici*) inoculum under a first wheat crop, named the take-all inoculum build-up (TAB) trait (McMillan *et al.*, 2011). However, there was no clear correspondence between the previously described TAB phenotypes of Cadenza and Hereward, low and high TAB, respectively, and their ability to support populations of *G. hyphopodioides* in this study (11.9% and 14.7% of roots colonized with *G. hyphopodioides* when baited with Hereward in the soil core bioassay; Supplementary Table S6)

Finally, the fourth aim was to establish whether there was any interaction between second wheat cultivar choice and level of root colonization by *G. hyphopodioides.* The majority of cultivars were found to support low levels of root colonization when the field plot cultivar represented the subsequent second wheat, rather than Hereward. However, significant interactions were also evident. Nine cultivars across the two years consistently exhibited the ability to support medium levels of *G. hyphopodioides* root colonization, independent of second wheat choice. For example, the elite cultivars Scout and KWS Kielder showed the highest level of *G. hyphopodioides* root colonization, regardless of the second wheat cultivar choice. On the one hand, the cultivar Alchemy consistently had the lowest level of *G. hyphopodioides* root colonization across the two second wheat cultivar choices, while on the other hand cultivars Zulu, Leeds, and KWS Croft indicated contrasting results from the two baiting methods.

Collectively, these data provide the first evidence for complex host genotype–*G. hyphopodioides* interactions occurring under both arable field conditions and in the 5 week seedling pot bioassay. The seedling pot bioassay screened a wide variety of cereal germplasm and cultivars, both modern and historical, yet there was little difference in the ability of *G. hyphopodioides* to colonize the roots of this diverse wheat germplasm under artificial conditions. The soil core bioassay from the experimental field trials screened less diverse modern wheat cultivars and revealed statistically significant differences in the ability of these cultivars to be colonized and also to support natural populations of *G. hyphopodioides* in the soil. These data suggest that wheat plants at the seedling stage may differ in their interaction with *G. hyphopodioides* during root colonization compared with adult plants in the field. The significantly different results obtained using the two baiting methods supports the suggestion that the presence of fungal inoculum (measured using Hereward as the baiting cultivar) is a trait independent of seedling root colonization. It is highly likely that the two phenomena are controlled by different mechanisms and may involve interactions with other soil-dwelling microbes and/or root exudates.

High extrapolated yield data were calculated for both field experiments (2015 range, 17.83–25.57 t ha^–1^; 2016 range, 12.68–22.93 t ha^–1^; Supplementary Table S9). There appears to be no strong evidence of a detrimental effect of *G. hyphopodioides* colonization on the yield of the plots. This complements field trials conducted in Australia investigating the cross-protection of *G. hyphopodioides* against take-all disease (Wong *et al.*, 1996).

One *G. hyphopodioides* isolate has been patented for take-all control in Australia (Wong *et al.*, 1996). No commercial use has been documented, and pelleting wheat seeds with *G. hyphopodioides* is not currently utilized as a method of biological control against take-all disease. The percentage of UK fields that contain this beneficial organism is unknown. However, this soil-borne species has been documented worldwide, including the USA, Australia, Poland, and Germany, and was identified in three of the four suppressive field sites on the arable farm used for this study. The ability of elite winter wheat cultivars to support and be colonized by natural populations of *G. hyphopodioides* under a first wheat crop suggests important host genotype–fungus interactions which, if harnessed, could potentially provide an additional management strategy, not only in the UK, to help combat take-all root disease in second wheats.

From a wheat breeding perspective, there does not appear to be any interaction between *G. hyphopodioides* root colonization and the National Association of British and Irish Flour Millers (nabim) groupings or pedigrees of the elite wheat cultivars. For example, the Robigus pedigree is found in several cultivars within the AHDB 2013/2014 RL and lines from the 2014/2015 RL winter wheat cultivars, yet there appear to be no similarities across these cultivars in their level of *G. hyphopodioides* root colonization with either of the second wheat cultivar choices. This suggests that the trait is not under simple genetic control and could also be influenced by environmental factors such as soil type and soil moisture, and biological factors such as the overall make-up of the rhizosphere/soil microbiome. However, consistent differences across the two field seasons were observed for a subset of nine cultivars, suggesting that suitable mapping populations could be generated to investigate the genetic basis of these interactions.

In summary, this is the first report of two robust field trial data sets that have revealed that UK elite winter wheat cultivars differ in their ability to support and be colonized by natural populations of the take-all root disease-suppressing fungus, *G. hyphopodioides*, under a first wheat crop. Although there were some clear inconsistencies between field seasons, this dual data set reveals that a subset of nine elite UK winter wheat cultivars consistently supported fungal inoculum and seedling root colonization by *G. hyphopodioides.* These cultivars have the potential to be used to encourage populations of introduced or resident beneficial fungi for the control of take-all disease in short wheat rotations. Further research is now required to explore the genetic and mechanistic basis of this interaction and the influence of environmental and genetic factors on soil population establishment, root colonization, and take-all control.

## Supplementary data

Supplementary data are available at *JXB* online.

Fig. S1. Previous field trial sites on the Rothamsted Farm that have shown suppression of take-all disease in experiments.

Table S1. Soil core sampling details for establishing the isolate collection.

Table S2. Experimental field trial details to evaluate the ability of elite UK winter wheat cultivars to support *Gaeumannomyces hyphopodioides* inoculum under a first wheat crop across the two field seasons.

Table S3. Details of fertilizer, pesticide, and growth regulator applications to the two experimental field trials.

Table S4. GenBank accession numbers for sequences used in the phylogenetic analysis.

Table S5. Percentage of roots colonized with *Gaeumannomyces hyphopodioides* in the soil core bioassay for the two field trials in 2015 and 2016.

Table S6. Combined REML variance components analysis of mean percentage of roots colonized with *Gaeumannomyces hyphopodioides* in the soil core bioassay for the two field trials in 2015 and 2016.

Table S7. Percentage of roots infected with take-all (*Gaeumannomyces tritici*) in the soil core bioassay for the two field trials in 2015 and 2016.

Table S8. Combined REML variance components analysis of mean percentage of roots infected with take-all (*Gaeumannomyces tritici*) in the soil core bioassay for the two field trials 2015 and 2016.

Table S9. Grain yield for elite wheat cultivars for each experimental field trial and mean grain yields across two field trials analysed by a combined REML variance components analysis.

Table S10. Monthly rainfall (mm) and maximum daily temperature (°C) for the months of May–August during the two field trial seasons of 2015 and 2016.

Supplementary Figure and Table
