# Supplementary material for: Elite UK winter wheat cultivars differ in their ability to support the colonization of beneficial root-infecting fungi
Source: J Exp Bot. 2018 Apr 10;69(12):3103–15. doi: 10.1093/jxb/ery136 (PMC5972604; doi:10.1093/jxb/ery136)
Supplement: Supplementary Figure and Table [file ery136_suppl_supplementary_materials.pdf]

Supplementary Data

Title: Elite UK winter wheat cultivars differ in their ability to support the colonization of beneficial root-infecting fungi

Sarah-Jane Osborne[1]*, Vanessa E. McMillan[1], Rodger White[2] and Kim E. Hammond-Kosack[1]†

[1]Department of Biointeractions and Crop Protection, Rothamsted Research, Harpenden, Hertfordshire, AL5 2JQ, UK

[2]Department of Computational and Analytical Systems, Rothamsted Research, Harpenden, Hertfordshire, AL5 2JQ, UK

*Current address: AHDB, Ashton House, Ambury Road South, Huntingdon, Cambridgeshire, UK, PE29 3EH

†Corresponding author: kim.hammond-kosack@rothamsted.ac.uk, Tel: +44(0) 1582 938240

Supplementary Fig. S1. Previous field trial sites on the Rothamsted Farm that have shown suppression of take-all disease in experiments

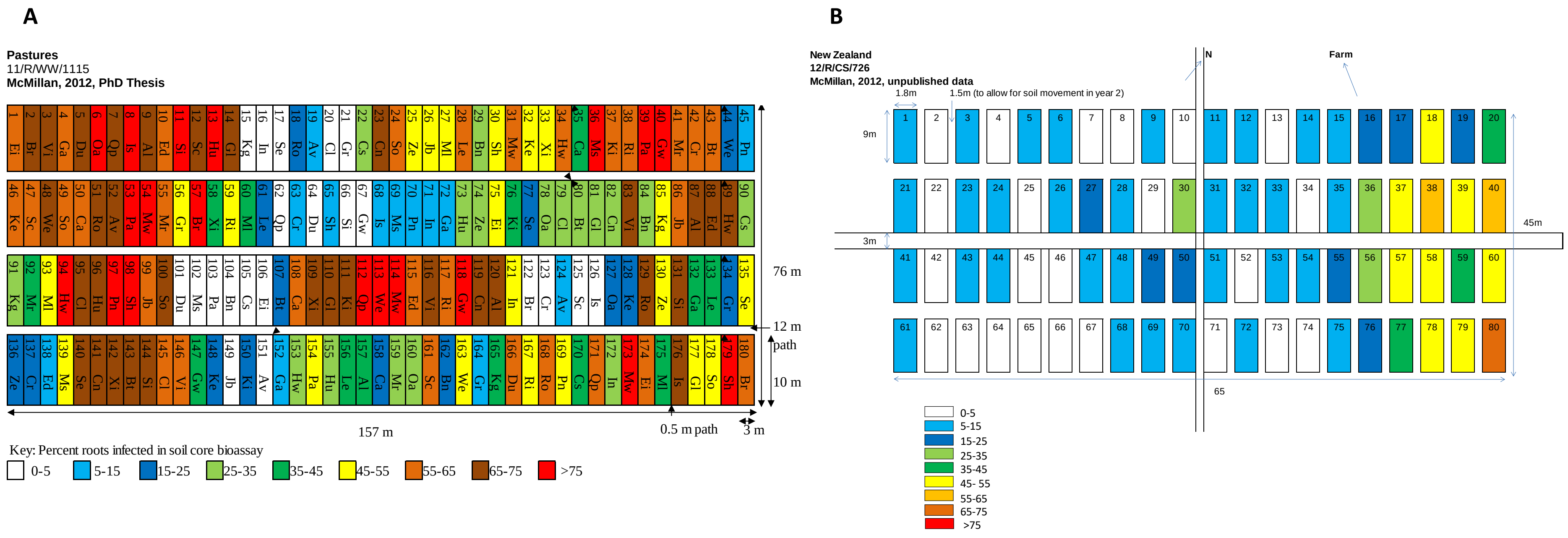

A. Pastures field trial 11/R/WW/1115, extremely patchy distribution of take-all disease. The soil core bioassay seedlings were assessed in 2011 and the characteristic subepidermal colonization phenotype of *G. hyphopodioides* and related Magnaporthaceae sp. was identified at moderate levels. This phenotype was typically associated with the bioassay plants showing a lack of black take-all lesions (McMillan, 2012, PhD thesis).

B. New Zealand field trial 12/R/CS/726, very low levels of take-all disease on the left-hand side of the field trial. This area corresponded with the characteristic subepidermal colonization phenotype of *G. hyphopodioides* and related Magnaporthaceae sp. being visible on seedling roots.

Summerdells I & II field trial 09/R/WW/901-2, WGIN Diversity trial, McMillan, 2009, unpublished data. Field trial blocks 1 and 2 of the WGIN Diversity experiment in Summerdells II had an average of 29% and 40% roots infected with take-all disease, respectively, in a post-harvest soil core bioassay. Whereas field trial block 3 of the experiment, in Summerdells I, had an average of only 15% roots infected with take-all, and the characteristic subepidermal colonization phenotype of *G. hyphopodioides* and related Magnaporthaceae sp. was visible on seedling roots.

Supplementary Table S1. Soil core sampling details for establishing the isolate collection

Rothamsted field	Sample date	Number of sampled cores to set up soil core bioassay	Crop and cultivar	Number of cores with visual *G. hyphopodioides* and related species phenotypes	Number of isolates obtained
Great Knott III	12.08.13	90	*Hordeum vulgare* stubble, Saffron	16/90	0
New Zealand	28.08.13	50	*Hordeum vulgare* stubble, Tipple	41/50	2
Pastures	03.09.13	100	*Triticum aestivum* stubble, Conqueror	77/100	5
Summerdells I	03.09.14	100	*Triticum aestivum* stubble, Conqueror	24/100	2

Supplementary Table S2. Experimental field trial details to evaluate the ability of elite UK winter wheat cultivars to support *Gaeumannomyces hyphopodioides* inoculum under a first wheat crop across the two field seasons.

Field season (field trial code)	Rothamsted field	Plot size (m)	Seed rate (seeds per m^2)	Sowing date	Harvest date	Sampling date for soil core bioassay
2015 (2015/R/WW/1516)	New Zealand	1 x 1	350	10/10/14	17/08/15	02-03/09/15
2016 (2016/R/WW/1620)	New Zealand	1 x 1	350	02/10/15	09/08/16	11-12/08/16

Supplementary Table S3. Details of fertiliser, pesticide and growth regulator applications to the two experimental field trials.

	Fertiliser and pesticide applications		
Field season	Date applied	Application	Product
2015	08/10/2015	Herbicides	Movon & Stomp Aqua
	23/10/2015	Insecticide	Hallmark Zeon
	03/11/2015	Insecticides	Crystal & Hallmark Zeon
	08/03/2016	Fertiliser	Doubletop
	01/04/2016	PGRs & fungicides	3C Chlormequat 750,
	04/04/2016	Fertiliser	Nitram
	19/04/2016	Herbicides	Chex & Pacifica
	26/04/2016	Fertiliser	Nitram
	05/05/2016	Herbicides & fungicide	Keystone NXT, Hatchet
	25/05/2016	Fungicides	VORTEX & Clayton
	09/07/2016	Fungicide & insecticide	Cello & Hallmark Zeon
2016	31/10/2014	Herbicides & fungicides	Liberator & Anthem
	20/11/2014	Insecticide	Hallmark Zeon
	12/03/2015	Fertiliser	DoubleTop
	25/03/2015	Fertiliser	Calcifert
	27/03/2015	Fungicides & PGRs	Artemis, Bravo 500 & 3C
	30/03/2015	Fertiliser	Nitram
	30/03/2015	Fertiliser	Nitram
	22/04/2015	Fungicides	Kingdom & Balear720
	01/05/2015	Fertiliser	Nitram
	22/05/2015	Herbicides & fungicides	Ally Max, Hatchet-Xtra &
	08/06/2015	Fungicide	Cello

Supplementary Table S4. GenBank accession numbers for sequences used in the phylogenetic analysis.

Fungal species and isolate identification	Country and host	GenBank accession number
Gaeumannomyces graminis CPC 26020[1]	USA, *Cynodon dactylon* x *C. transvaalensis*	KX306498.1
Gaeumannomyces graminis CPC 26027[1]	USA, *Cynodon dactylon* x *C. transvaalensis*	KX306499.1
Gaeumannomyces graminis CPC 26029[1]	USA, *Cynodon dactylon* x *C. transvaalensis*	KX306500.1
Gaeumannomyces graminis CPC 26033[1]	USA, *Cynodon dactylon* x *C. transvaalensis*	KX306501.1
Gaeumannomyces graminis CPC 26035[1]	USA, *Cynodon dactylon* x *C. transvaalensis*	KX306502.1
Gaeumannomyces graminis CPC 26039[1]	USA, *Cynodon dactylon* x *C. transvaalensis*	KX306503.1
Gaeumannomyces graminis CPC 26042[1]	USA, *Cynodon dactylon* x *C. transvaalensis*	KX306504.1
Gaeumannomyces graminis CPC 26045[1]	USA, *Cynodon dactylon* x *C. transvaalensis*	KX306505.1
Gaeumannomyces hyphopodioides CBS 350.77[1, †]	UK, *Zea mays*	KX306506.1
Gaeumannomyces hyphopodioides CBS 541.86[1]	Germany, *Triticum aestivum*	KX306507.1
Gaeumannomyces hyphopodioides CPC 26247[1]	UK, *Triticum aestivum*	KX306508.1
Gaeumannomyces hyphopodioides CPC 26248[1]	UK, *Triticum aestivum*	KX306509.1
Gaeumannomyces hyphopodioides CPC 26249[1]	UK, *Triticum aestivum*	KX306510.1
Gaeumannomyces hyphopodioides CPC 26252[1]	Poland, *Triticum aestivum*	KX306512.1
Gaeumannomyces hyphopodioides CPC 26264[1]	UK, *Triticum aestivum*	KX306513.1
Gaeumannomyces hyphopodioides CPC 26265[1]	UK, *Triticum aestivum*	KX306514.1
Gaeumannomyces hyphopodioides CPC 26267[1]	Australia, *Pennisetum clandestinum*	KX306515.1
Gaeumannomyces tritici CBS 247.29[1]	Netherlands, *Triticum* sp.	KM484839.1
Gaeumannomyces tritici CBS 249.29[1]	-, *Triticum aestivum*	KM484840.1
Gaeumannomyces tritici CBS 905.73[1]	Australia, *Triticum aestivum*	KM484841.1
Gaeumannomyces tritici CBS 131293[1]	USA, *Triticum* sp.	KX306526.1
Gaeumannomyces tritici CPC 26069[1]	USA, -	KX306527.1
Gaeumannomyces tritici CPC 26277[1]	UK, *Elymus repens*	KX306537.1
Gaeumannomyces tritici CPC 26282[1]	UK, *Triticum aestivum*	KX306541.1
Gaeumannomyces tritici CPC 26283[1]	UK, *Triticum aestivum*	KX306542.1
Gaeumannomyces tritici R3-111a-1[2]	USA, *Triticum aestivum*	FJ541430.1
Magnaporthaceae sp. CPC 26284[1]	UK, *Triticum aestivum*	KX306546.1
Magnaporthaceae sp. K8[3]	Germany, *Triticum aestivum*	AJ132541.1
Magnaporthaceae sp. 437[3]	Germany, *Hordeum vulgare*	AJ132542.1
Pyricularia grisea BR0029[1]	Brazil, *Digitaria sanguinalis*	KM484880.1
Pyricularia grisea CR0024[1]	South Korea, *Lolium perenne*	KM484882.1
Uncultured *Phialophora* SMOTU257[4]	Germany, *Zea mays*	HG937137.1

[1] Hernández-Restrepo et al. (2016).
[2] from Kwak et al. (2010).
[3] Ulrich et al. (2000).
[4] Moll et al. (2016).
† Type material.

Supplementary Table S5. Percentage of roots colonized with *Gaeumannomyces hyphopodioides* in the soil core bioassay for the two field trials in 2015 and 2016.

	Logit % of roots colonized with *G. hyphopodioides* (back-transformed means)							
	Baited with Hereward		Baited with field plot cultivar		Baited with Hereward		Baited with field plot cultivar	
Cultivar	2015				2016			
Alchemy	-1.755	(2.40)	-2.260	(0.58)	-1.532	(4.46)	-2.355	(0.89)
Beluga	-1.575	(3.61)	-2.197	(0.72)	-0.914	(13.85)*	-2.599	(0.55)
Chilton	-1.469	(4.53)*	-2.360	(0.38)	-0.756	(18.05)*	-2.400	(0.82)
Denman	-1.425	(4.97)	-2.074	(1.06)	-0.796	(16.92)*	-2.546	(0.61)
Gallant	-1.407	(5.16)	-1.419	(5.03)	-0.795	16.94)	-0.912	(13.89)
Delphi	-1.391	(5.33)*	-2.382	(0.35)	-0.759	(17.99)*	-2.188	(1.24)
Einstein	-1.382	(5.43)	-1.056	(10.28)†	-0.747	(18.33)	-0.760	(17.96)
Cordiale	-1.370	(5.57)	-1.040	(10.61)†	-0.733	(18.75)	-0.504	(26.74)†
Dickens	-1.365	(5.63)	-1.780	(2.26)	-1.400	(5.74)	-2.111	(1.44)
Viscount	-1.340	(5.92)*	-2.300	(0.50)	-0.976	(12.44)*	-2.604	(0.55)
Cougar	-1.324	(6.11)*	-2.269	(0.56)	-1.347	(6.33)	-2.479	(0.70)
Cadenza	-1.318	(6.18)	-1.356	(5.73)	-0.856	(15.30)	-0.844	(14.58)
Grafton	-1.299	(6.43)	-1.771	(2.31)	-0.565	(24.40)	-0.506	(26.66)†
JB Diego	-1.289	(6.55)	-0.905	(13.56)†	-0.713	(19.36)	-0.733	(18.74)
Conqueror	-1.276	(6.74)	-2.120	(0.92)	-1.259	(7.45)	-2.167	(1.29)
Crusoe	-1.251	(7.07)	-2.094	(1.00)	-0.962	(12.74)*	-2.290	(1.01)
KWS Santiago	-1.248	(7.11)	-2.082	(1.03)	-0.934	(13.38)*	-2.263	(1.07)
KWS Podium	-1.238	(7.25)	-1.520	(4.07)	-0.933	(13.41)	-0.803	(16.70)†
Relay	-1.202	(7.78)	-1.089	(9.67)	-0.619	(22.48)	-1.156	(9.01)
Solstice	-1.195	(7.89)	-1.021	(10.99)†	-0.748	(18.31)	-0.777	(17.45)
KWS Gator	-1.188	(8.01)	-1.333	(6.00)	-0.804	(16.70)	-0.847	(15.53)
Invicta	-1.169	(8.30)*	-2.277	(0.54)	-0.632	(22.02)*	-2.257	(1.08)
Duxford	-1.156	(8.52)	-1.253	(7.04)	-0.868	(14.98)	-1.151	(9.09)
Claire	-1.153	(8.55)*	-2.453	(0.24)	-0.797	(16.88)*	-2.531	(0.63)
Myriad	-1.125	(9.04)	-2.117	(0.93)	-1.064	(10.64)	-2.121	(1.42)
Horatio	-1.110	(9.21)*	-2.349	(0.40)	-1.334	(6.49)	-2.438	(0.76)
Hereward	-1.109	(9.31)	-1.134	(8.88)	-0.771	(17.61)	-0.458	(28.56)†
Evolution	-1.106	(9.37)	-1.702	(2.72)	-0.929	(13.50)	-2.111	(1.45)
Panorama	-1.086	(9.72)	-1.333	(5.99)	-0.667	(20.83)	-0.870	(14.93)
KWS Target	-1.062	(10.17)	-1.834	(1.99)	-1.222	(7.98)	-2.400	(0.82)
Scout	-1.032	(10.76)	-0.859	(14.71)†	-0.629	(22.13)	-0.529	(25.78)†
Tuxedo	-0.996	(11.51)	-1.721	(2.60)	-1.171	(8.76)*	-2.418	(0.79)
Revelation	-0.949	(12.53)*	-2.017	(1.24)	-1.287	(7.09)*	-2.556	(0.60)
KWS Kielder	-0.923	(13.15)	-1.043	(10.55)	-0.528	(25.79)	-0.614	(22.64)
Monterey	-0.906	(13.54)	-1.902	(1.68)	-1.210	(8.17)	-2.313	(0.97)
KWS Sterling	-0.887	(13.99)*	-2.191	(0.73)	-0.887	(14.51)*	-2.388	(0.84)
KWS Croft	-0.851	(14.92)*	-2.067	(1.08)	-0.852	(15.38)*	-2.340	(0.92)
Zulu	-0.828	(15.53)	-1.802	(2.15)	-0.699	(19.80)*	-2.493	(0.68)
Cocoon	-0.824	(15.64)*	-2.167	(0.79)	-1.196	(8.37)	-2.295	(1.01)
Leeds	-0.701	(19.25)*	-2.116	(0.93)	-1.051	(10.88)	-2.049	(1.63)
df	39				39			
SED (logit scale)	0.231				0.194			
F statistic	5.58				13.5			
F probability (interaction effect)	<0.001				<0.001			
Grand mean	-1.769	3.82	-1.182	8.72	-1.754	7.55	-0.924	14.63

Very low root colonization: <5%, low root colonization: 5-10%, medium root colonization: >10%.

* Percentage of roots colonized when baited with Hereward is 10% + of the percentage of roots colonized when baited with the field plot cultivar.

† Percentage of roots colonized when baited with the field plot cultivar is higher than the percentage of roots colonized when baited with Hereward.

Supplementary Table S6. Combined REML variance components analysis of mean percentage of roots colonized with *Gaeumannomyces hyphopodioides* in the soil core bioassay for the two field trials in 2015 and 2016.

	Logit % of roots colonized with *G. hyphopodioides* (back-transformed means)			
Cultivar	Baited with Hereward		Baited with field plot cultivar	
Alchemy	-1.557	(4.25)	-2.313	(0.97)
Dickens	-1.320	(6.66)	-1.984	(1.86)
Cougar	-1.263	(7.40)	-2.399	(0.82)
Conqueror	-1.189	(8.49)	-2.169	(1.29)
Horatio	-1.128	(9.48)	-2.387	(0.84)
Beluga	-1.117	(9.67)	-2.349	(0.90)
Viscount	-1.059	(10.73)	-2.448	(0.74)
Myriad	-1.051	(10.90)	-2.144	(1.36)
KWS Target	-1.045	(11.01)	-2.102	(1.47)
Crusoe	-1.033	(11.25)	-2.184	(1.25)
Revelation	-1.022	(11.47)	-2.248	(1.10)
Monterey	-1.021	(11.48)	-2.135	(1.38)
Chilton	-1.011	(11.70)	-2.367	(0.87)
Tuxedo	-1.009	(11.73)	-2.055	(1.61)
KWS Santiago	-1.007	(11.78)	-2.168	(1.29)
KWS Podium	-1.006	(11.80)	-1.148	(9.15)
Cadenza	-1.002	(11.88)	-1.119	(9.64)
Denman	-0.966	(12.66)	-2.257	(1.08)
Gallant	-0.965	(12.68)	-1.105	(9.89)
Einstein	-0.958	(12.83)	-0.902	(14.14)
Evolution	-0.957	(12.85)	-1.934	(2.05)
Cordiale	-0.956	(12.88)	-0.766	(17.76)
Delphi	-0.940	(13.23)	-2.251	(1.10)
Duxford	-0.928	(13.52)	-1.202	(8.28)
Solstice	-0.921	(13.69)	-0.930	(13.48)
JB Diego	-0.916	(13.80)	-0.825	(16.12)
Cocoon	-0.915	(13.83)	-2.214	(1.18)
KWS Gator	-0.889	(14.47)	-1.062	(10.67)
Hereward	-0.881	(14.65)	-0.850	(15.45)
Invicta	-0.858	(15.24)	-2.294	(1.01)
Claire	-0.847	(15.53)	-2.455	(0.73)
Grafton	-0.841	(15.68)	-1.112	(9.76)
Leeds	-0.832	(15.91)	-2.117	(1.43)
Relay	-0.806	(16.62)	-1.132	(9.42)
KWS Sterling	-0.799	(16.82)	-2.264	(1.07)
Panorama	-0.762	(17.90)	-1.075	(10.44)
KWS Croft	-0.742	(18.50)	-2.210	(1.19)
Scout	-0.722	(19.11)	-0.675	(20.60)
Zulu	-0.696	(19.90)	-2.157	(1.32)
KWS Kielder	-0.599	(23.18)	-0.796	(16.92)
df	39			
SED (logit scale)	0.171			
Wald statistic	637.76			
F Probability (interaction effect)	<0.001			

Very low root colonization: <5%, low root colonization: 5-10%, medium root colonization: >10%.

Supplementary Table S7. Percentage of roots infected with take-all (*Gaeumannomyces tritici*) in the soil core bioassay for the two field trials in 2015 and 2016.

	Logit % of roots infected with *G. tritici* (back-transformed means)			
Cultivar	2015		2016	
Grafton	-2.697	(0.00)	-2.596	(0.55)
Delphi	-2.623	(0.02)	-2.229	(1.15)
Leeds	-2.615	(0.03)	-2.359	(0.89)
Crusoe	-2.591	(0.06)	-2.482	(0.69)
Beluga	-2.579	(0.07)	-2.568	(0.59)
Invicta	-2.539	(0.12)	-2.590	(0.56)
KWS Target	-2.538	(0.12)	-2.006	(1.78)
Einstein	-2.540	(0.12)	-2.429	(0.77)
Cordiale	-2.537	(0.12)	-2.466	(0.72)
Chilton	-2.514	(0.15)	-2.387	(0.84)
Gallant	-2.518	(0.15)	-2.168	(1.29)
KWS Santiago	-2.507	(0.16)	-2.471	(0.71)
KWS Stirling	-2.509	(0.16)	-2.569	(0.58)
Cocoon	-2.471	(0.21)	-1.928	(2.07)
Zulu	-2.463	(0.22)	-2.245	(1.11)
Panorama	-2.465	(0.22)	-2.275	(1.05)
KWS Podium	-2.436	(0.26)	-2.216	(1.18)
Monterey	-2.431	(0.27)	-2.223	(1.16)
Scout	-2.421	(0.28)	-2.235	(1.13)
KWS Gator	-2.403	(0.31)	-2.447	(0.74)
Claire	-2.369	(0.37)	-2.086	(1.52)
Horatio	-2.365	(0.37)	-2.470	(0.71)
JB Diego	-2.368	(0.37)	-2.474	(0.71)
Revelation	-2.364	(0.38)	-2.362	(0.88)
Denman	-2.356	(0.39)	-2.364	(0.88)
Conqueror	-2.351	(0.40)	-2.080	(1.54)
Cougar	-2.323	(0.45)	-2.198	(1.22)
Solstice	-2.323	(0.45)	-2.360	(0.88)
Myriad	-2.286	(0.52)	-2.272	(1.05)
Duxford	-2.260	(0.58)	-2.251	(1.10)
KWS Croft	-2.251	(0.60)	-2.109	(1.45)
KWS Kielder	-2.250	(0.60)	-2.179	(1.26)
Viscount	-2.229	(0.65)	-2.455	(0.73)
Evolution	-2.225	(0.65)	-2.297	(1.00)
Tuxedo	-2.221	(0.66)	-2.248	(1.10)
Hereward	-2.206	(0.70)	-2.383	(0.85)
Dickens	-2.111	(0.95)	-2.203	(1.21)
Cadenza	-2.107	(0.96)	-2.203	(1.21)
Alchemy	-2.048	(1.14)	-2.351	(0.90)
Relay	-1.985	(1.35)	-2.422	(0.78)
df	39		39	
SED (logit scale)	0.169		0.186	
Wald statistic	75.270		-	
F statistic	-		1.50	
F probabilty	<0.001		0.045	

Supplementary Table S8. Combined REML variance components analysis of mean percentage of roots infected with take-all (*Gaeumannomyces tritici*) in the soil core bioassay for the two field trials 2015 and 2016.

	Logit % of roots infected with *G. tritici* (back-transformed means)			
	Baited with Hereward		Baited with field plot cultivar	
KWS Sterling	-2.655	(0.49)	-2.366	(0.87)
Beluga	-2.644	(0.50)	-2.519	(0.65)
Grafton	-2.593	(0.56)	-2.650	(0.50)
Cordiale	-2.584	(0.57)	-2.456	(0.73)
Delphi	-2.579	(0.57)	-2.343	(0.91)
KWS Santiago	-2.515	(0.65)	-2.436	(0.76)
Leeds	-2.512	(0.65)	-2.469	(0.71)
Crusoe	-2.498	(0.67)	-2.585	(0.57)
Zulu	-2.486	(0.69)	-2.262	(1.07)
Claire	-2.479	(0.70)	-1.989	(1.84)
Einstein	-2.478	(0.70)	-2.494	(0.68)
Denman	-2.470	(0.71)	-2.290	(1.02)
Invicta	-2.469	(0.71)	-2.616	(0.53)
KWS Gator	-2.459	(0.73)	-2.401	(0.81)
Viscount	-2.436	(0.76)	-2.182	(1.26)
Revelation	-2.412	(0.80)	-2.275	(1.05)
Gallant	-2.409	(0.80)	-2.287	(1.02)
JB Diego	-2.403	(0.81)	-2.435	(0.76)
Cocoon	-2.398	(0.82)	-1.996	(1.81)
Horatio	-2.382	(0.85)	-2.457	(0.73)
Scout	-2.367	(0.87)	-2.310	(0.98)
Monterey	-2.354	(0.89)	-2.291	(1.01)
Evolution	-2.340	(0.92)	-2.195	(1.23)
Solstice	-2.338	(0.92)	-2.351	(0.90)
KWS Podium	-2.328	(0.94)	-2.357	(0.89)
Chilton	-2.325	(0.95)	-2.560	(0.59)
Panorama	-2.320	(0.96)	-2.411	(0.80)
KWS Target	-2.318	(0.96)	-2.285	(1.03)
KWS Croft	-2.259	(1.08)	-2.122	(1.42)
Cougar	-2.238	(1.13)	-2.337	(0.92)
Myriad	-2.223	(1.16)	-2.289	(1.02)
Duxford	-2.196	(1.22)	-2.326	(0.95)
Hereward	-2.188	(1.24)	-2.406	(0.81)
Relay	-2.185	(1.25)	-2.208	(1.20)
KWS Kielder	-2.176	(1.27)	-2.235	(1.13)
Tuxedo	-2.135	(1.38)	-2.326	(0.95)
Cadenza	-2.068	(1.57)	-2.220	(1.17)
Dickens	-2.035	(1.68)	-2.279	(1.04)
Conqueror	-1.950	(1.99)	-2.539	(0.62)
Alchemy	-1.949	(1.99)	-2.387	(0.84)
df	39			
SED (logit scale)	0.1816			
F probability (interaction effect)	0.048			
F statistic	1.41			

Supplementary Table S9. Grain yield for elite wheat cultivars for each experimental field trial and mean grain yields across two field trials analysed by a combined REML variance components analysis.

Cultivar	Grain yield[1] (tonnes/hectare)		
	2015	2016	Combined
KWS Sterling[2]	17.83	12.68	15.08
Solstice	18.30	17.98	18.31
Hereward	19.08	16.84	18.04
KWS Podium	20.30	15.55	17.88
Cadenza	20.48	17.89	19.19
KWS Target	20.79	16.35	18.38
Beluga	21.21	18.88	20.11
Chilton	21.34	17.28	19.03
Scout	21.48	18.39	20.02
Monterey	21.60	19.18	20.36
KWS Croft	21.72	20.98	21.42
Gallant	22.05	18.92	20.56
Cordiale	22.06	19.17	20.66
Denman	22.28	20.20	21.40
Grafton	22.29	20.10	21.26
Cougar	22.67	20.58	21.78
Revelation	22.70	19.83	21.21
Myriad	22.71	20.33	21.45
JB Diego	22.84	21.79	22.42
Viscount	22.88	19.71	21.27
Tuxedo	22.93	20.37	21.80
Einstein	22.95	21.21	22.11
Leeds	23.09	20.89	22.00
Dickens	23.13	22.22	22.77
Claire	23.17	20.60	21.68
Delphi	23.19	20.13	21.67
Conqueror	23.53	21.68	22.62
Invicta	23.64	19.35	21.37
Panorama	23.68	19.31	21.36
Horatio	23.72	20.75	22.32
KWS Gator	24.00	20.75	22.27
Zulu	24.00	21.56	22.82
Alchemy	24.05	22.55	23.38
Relay	24.05	21.96	23.11
Cocoon	24.12	21.17	22.74
Evolution	24.31	22.08	23.29
Duxford	24.32	20.46	22.39
Crusoe	24.76	21.99	23.39
KWS Santiago	24.95	22.93	23.98
KWS Kielder	25.57	20.25	22.71
df	39	39	39
SED	0.824	0.784	0.599
F statistic	9.69	14.91	20.00
F probability	<0.001	<0.001	<0.001

[1] Field trial plots were combine-harvested at grain maturity and fresh grain weights recorded. A subsample of grain (80 g) from each plot was oven-dried for 16 hours at 105°C. Grain yields were adjusted to 85% dry matter and scaled to tonnes/ha.

[2] Poor germination in the field.

Supplementary Table S10. Monthly rainfall (mm) and maximum daily temperature (°C) for the months of May-August during the two field trial seasons of 2015 and 2016 (data downloaded from the electronic Rothamsted Archive (e-RA)).

Year					
Rainfall (mm)	May	June	July	August	Total
2015	68.5	26.8	132.6	83.2	311.1
2016	39.3	84.6	27.0	30.1	181.0
Maximum temperature (°C)					Average
2015	15.6	19.5	21.4	20.5	19.3
2016	17.4	19.0	22.2	22.7	20.3
